# Role of regulatory capacity in the animal and human health systems in driving response to zoonotic disease outbreaks in the the Mekong region

**DOI:** 10.1016/j.onehlt.2022.100369

**Published:** 2022-01-10

**Authors:** Barbara McPake, Katherine Gilbert, Sreytouch Vong, Bandeth Ros, Phalmony Has, Anh Tuan Khuong, Pham-Duc Phuc, Quoc Cuong Hoang, Duc Hai Nguyen, Latsamy Siengsounthone, Chanthaly Luangphaxay, Peter Annear, Justin McKinley

**Affiliations:** a Nossal Institute for Global Health, Melbourne, Australia; b Independent consultants contracted by the Nossal Institute for Global Health, Phnom Penh, Cambodia; c National Institute of Public Health, Phnom Penh, Cambodia; d Health Strategy and Policy Institute, Hanoi, Viet Nam; e Center for Public Health and Ecosystem Research, Hanoi University of Public Health, Hanoi, Viet Nam; f Pasteur Institute Ho Chi Minh City, Ho Chi Minh City, Viet Nam; g Lao Tropical and Public Health Institute, Vientiane Capital, Laos

**Keywords:** Animal health, Avian influenza, Human health, Mekong, One health

## Abstract

We conducted a policy situation analysis in three Mekong region countries, focused on how the animal and human health systems interact to control avian influenza (AI). The study used scoping literature reviews aimed at establishing existing knowledge concerning the regulatory context. We then conducted a series of key informant interviews with national and sub-national government officials and representatives of producers and poultry farmers to understand their realities in managing the complex interface of the two sectors to control AI.

We found signs of formal progress in establishing the policy and legislative frameworks needed to enable cooperation of the two sectors but a series of constraints that impede their effective operation. These included the competitive relationships involved, especially with budgetary allocations and mandates that can conflict with each other. Many local actors also view development partners (e.g., bilateral and multilateral donors) as having a dominant role in establishing these collaborations, limiting the extent to which there is local ownership of the agenda.

The animal and human health sectors are not equally resourced, with the animal health sector disadvantaged in terms of surveillance and laboratory systems, human resources and financial allocations. Contrasting strategies for achieving objectives have also characterised the two sectors in recent decades, seeing a major shift towards the use of incentive-based approaches in the human health sector but very little parallel development in the animal health sector, largely dependent on command and control approaches.

Successful future collaborations between the two sectors are likely to depend on better resourcing in the animal health sector, increasing local ownership of the agenda, and ensuring that both sectors can use the full range of regulatory strategies available to achieve objectives.

## Highlights

• One health is impeded by low-level trust between animal and human health sectors.
• Bureaucratic red tape makes information sharing difficult between the two sectors.
• Both sectors are establishing legislative and policy frameworks zoonotic outbreaks
• Animal health is less resourced for service delivery, surveillance, etc.
• Human health sector incentivizes the market, animal health uses command and control.

## 1 Introduction

### 1.1 Avian influenza in the Mekong region and regulatory capacity

Governments in Cambodia, Lao PDR (henceforth Laos) and Vietnam have given attention to policies that promote protection against Highly Pathogenic Avian Influenza (HPAI) in the past 20 years. HPAI is of concern amongst emerging infectious diseases because its global case fatality rate in humans has averaged 53% since 2003 [1]. In the same period, case fatality rates in the countries of our study have been 66% in Cambodia (56 cases reported), 66% in Laos (three cases reported), and 50% in Vietnam (127 cases reported) [1]. Hence, should an outbreak become an epidemic or pandemic, the consequences would be severe.

The *Terrestrial and Aquatic Animal Health Codes* (‘the Animal Codes’) overseen by the World Organisation for Animal Health (OIE) and the International Health Regulations (IHR) overseen by WHO require states to take measures to prevent, detect and respond to outbreaks of avian influenza and other zoonotic infections. Such measures are dependent upon “*functional capacities in the animal and public health sector and collaboration, coordination and communication between them*” ([2], page 30). Guidance on the implementation of the IHR and Animal Codes originally envisaged short timeframes, which have come and gone, for the development of these critical capacities within the human and animal health systems to implement these measures [3].

Assessments of state capacities to implement the Animal Codes and IHR take place through voluntary Performance of Veterinary Services (PVS) Evaluation and Gap Analysis, and the Joint External Evaluation (JEE) of the IHR. The PVS, which assesses countries' capacity to implement legislation and regulation, ranked Cambodia and Laos at the level of one out of five concerning their implementation of regulations, meaning that “the [veterinary services] have no or very limited programmes or activities to ensure stakeholder compliance with relevant legislation and regulations” [4,5]. Vietnam received higher marks in its PVS for the implementation and regulation of legislation, although there were still shortcomings [6] and the JEE considered that animal health laws related to the IHR were not being complied with [7]. The lack of enforcement of existing regulations concerning drug supply is also a common theme in both the human and animal health literature in the three countries [4,5,8].

The WHO and OIE have recognised a need to move “*away from externally driven, short-term, emergency response type ‘vertical’ approaches*,” towards “*a more sustainable, ‘horizontal approach’ and long-term strengthening of*” animal and human health system*s* ([9], page 6). Regulation is considered a key policy intervention within systems strengthening, yet understanding of how regulatory capacities within animal and human health systems impact on collaboration between the two sectors and zoonotic disease prevention and response, is limited. Animal and human health systems share similar components or “building blocks” – they typically have a government ministry responsible for regulating public and private services, including the health workers and pharmaceuticals upon which clinical care is based, and the information generated through the system [10]. There has been a considerable body of work in the health system on how building regulatory capacity to adopt new approaches can create incentives for system wide improvements for access to, and quality of services [[11], [12], [13]], but understanding and comparison of capacities and approaches within animal health systems is lacking, and there is a gap in understanding how this impacts collaboration with respect to zoonotic disease responses.

This paper seeks to explore different regulatory capacities in the animal and human health systems, and how these impact zoonotic disease responses using Avian Influenza (AI) in the Mekong as a case study. We explored the role of both animal and human health systems in responding to zoonotic disease outbreaks, with a focus on regulatory policies that promote (1) timely notification of diseases and (2) early investment in preventative measures. In each of three countries, we aimed to understand (a) the existing regulatory capacities and strategies within the animal and human health sectors to implement the relevant international regulations, and (b) how these capacities shaped responses to AI. Existing conceptual frameworks informed our approach to this enquiry concerning regulatory capacity and collaboration, and are discussed below.

### 1.2 Concepts and context of regulation

Definitions of regulation vary in the breadth of action they encompass and the extent to which they recognise the role of non-state actors engaged in regulatory activity. This study adopts the ‘mid-way’ definition from Ensor and Weinzierl [11] :, *“regulation based on purposive actions initiated, although not necessarily implemented, by Government to address failures in the existing public and private [human or animal] health care system and promote current policy objectives”.* In other words, regulation addresses recognised market failures (e.g., public good problems) within the human or animal health care systems to enable the systems to achieve government set public health or veterinary goals. While derived for the human health system, it seems equally applicable to the animal health system.

The regulations of interest in this case study are those related to the objective of promoting early notification and response to zoonotic disease outbreaks. Drawing on the WHO Handbook for integrating the PVS into the JEE [14], this includes the following shared objectives across the human and animal health systems:

• Access to and quality of services, including actions to regulate the supply of services by public and private providers and to promote demand.
• Effective supply chains delivering pharmaceuticals for the prevention, treatment and control of zoonotic disease outbreaks to service providers.
• Surveillance and reporting of zoonotic disease, e.g., actions to regulate mandatory reporting of zoonotic disease outbreaks by public and private providers, and livestock owners.
• Emergency management (containment), including through quarantine and culling.

There is a range of regulatory actions that states can take to achieve the above objectives, from command and control approaches that require state enforcement of sanctions, to more market or incentive-based approaches that depend on the state to negotiate effective incentives and monitor outcomes [11].

Over the past three decades, Cambodia, Laos and Vietnam have each been impacted by similar waves of liberalisation, and have evolved from centrally planned, communist systems to decentralised market economies [[15], [16], [17]]. These changes have led to increased movement and trade of livestock locally and abroad, resulting in more frequent zoonotic disease outbreaks [18].

Concurrently, these changes have seen a significant transition in the human and animal health systems, with increasing reliance on private financing and private provision of health and veterinary care services. While there are limited data on service providers across both systems, it is evident that private provision is now substantial in each of the three countries. For example, with respect to the human health system, the private sector provides approximately 85% of primary health care in Cambodia [19] and 60% of outpatient care in Vietnam [20]. In Laos, while the government was the sole provider of veterinary drugs 30 years ago, its share has fallen to an estimated 33% of market share more recently [4].

Given the shared challenges of deregulation and increasing private sector involvement, the two sectors in all three countries now face similar challenges regarding how best to use regulatory tools to promote equitable access to quality services and pharmaceuticals in a mixed public-private system, as well as the shared challenge as to how best to respond to zoonotic disease outbreaks.

Dubash and Morgan [21] analyse regulation as a form of collaboration. They argue that literature on state capacities has primarily focused on “thin capacities” (e.g., autonomy, staffing and financial sustainability), whereas “thicker capacities” are needed to engage with the regulatory society - interested state and non-state actors - while maintaining procedural correctness, independence and reasoning. These thicker capacities resonate with the literature on zoonotic disease prevention and response, given the need for the animal and human health sectors to “collaborate, coordinate and communicate” in relation to zoonotic disease control. In a comparative case study on stewardship of zoonotic disease prevention and response in Indonesia and Thailand, Hort et al. [22] find that neither country has reached the point at which decisions in the event of an outbreak are seen as “credible, legitimate, and trusted by the general public” implying similar weaknesses of thicker capacities.

We apply this description of “thicker” capacities in our analysis of regulatory capacity, with a focus on relationships between the animal and human health systems and relationships with interested parties, being health workers who are subject to regulation and livestock owners.

## 2 Methods

This research project uses AI as a case study to understand the regulatory policies in place and the extent of implementation and collaboration across the animal and human health systems concerning (i) early reporting of flu-like illness and (ii) rapid containment in Cambodia, Laos, and Vietnam.

The aims of the project were to understand:

1. The regulatory capacities within the animal and human health systems; and
2. How these capacities shaped responses to AI, with a focus on timely notification of AI or HPAI and investment in preventative measures.

In addition, we sought to share results and experiences with partners and decision-makers across the human and animal health systems to make meaning of the data and together identify questions for future research.

The research design included:

1. Analysis of regulatory capacity relating to early prevention and response to zoonosis based on the most recent WHO [2] JEE and the OIE [23] PVS assessments in Cambodia, Laos and Vietnam.
2. Key informant interviews with national and sub-national government officials and representatives of producers/poultry farmers in Cambodia, Laos and Vietnam (interview guides are available in Appendices A–C).
3. A regional workshop to discuss and refine results in Vietnam (September 2019).

We obtained ethics approval from the University of Melbourne (1,954,014.1) and the Health Ethics Review Boards in Cambodia (no.112.NECHR), Laos (2019.42.MC) and Vietnam (563/QD-PAS).

### 2.1 Analysis of regulatory capacity

A literature review established that the regulatory capacities in the three countries were the subject of limited published research, and that the most in depth analysis was found within the WHO [2] JEE and the OIE [23] PVS assessments. We extracted data from these reports to the Dubash and Morgan [21] framework of thick and thin capacities, as shown in Table 1. Both the PVS and JEE define the capacities needed to implement the OIE animal codes and International Health Regulation (IHR) – referred to as critical competencies in the PVS. The PVS and the JEE use a rubric to define five capacity levels concerning each component, with a score from 1 to 5 (lowest to highest capacity) assigned. Generally, the PVS provides more of a qualitative description of the extent to which the capacity is fulfilled compared to the JEE and thus offers more insight for this review. The PVS notes where competency rankings declined or improved, but does not systematically explore reasons for capacity changes in the animal health sector. Therefore, the tool is less useful for understanding changes and influences on capacity. The PVS also explores the animal health sector in full. Conversely, the JEE focuses specifically on implementing the IHR, and thus the description of the human health sector is limited. We also used the ‘Health in Transition’ (HiT) documents produced by the Asia Pacific Observatory on Health Systems and Policies for Cambodia and Laos to supplement understanding of the human health sector in these two countries (no HiT is available in Vietnam).

**Table 1:** Mapping measures of the components and level of regulatory capacity against indicators within the PVS and JEE.

	PVS critical competencies	IHR JEE indicators
Engage with state and non-state actors	Coordination (internal, i.e.: within the veterinary service including public and private providers) (I-6A)	Coordination for IHR implementation (relates to multi-sectoral coordination) (P.2.1)
	Coordination (external) (I—6B)	Mechanisms for responding to zoonotic disease outbreaks (P.4.3)
	Communications (III-1)	Veterinarians or Animal Health Workforce (P 4.2)
	Staffing for the VS (I.1a-I.2a/b)	

* New indicator in the 2018 JEE guidance so it has not been considered in the most recent country assessments; ** refers to generalised enforcement; there are several specific regulatory areas covered in the PVS and JEE, which will not be considered in this review, due to scope.

### 2.2 Avian influenza case study

Researchers interviewed national and sub-national officials from the Ministries of Health and Agriculture in each country using purposive sampling, targeting officials responsible for AI or zoonoses (May–September 2019). We conducted interviews with poultry owners utilising a convenience sample targeting smallholder poultry owners in areas with a recent outbreak of AI in Cambodia and Vietnam. In Laos, large-scale poultry farmers, often without experience of an outbreak, were targeted.

Interview guides were drafted by the authors and revised in collaboration with research partners in each of the three countries. Regulation was explored through reference to policy and policy implementation in key areas related to the notification and timely notification of AI or HPAI and investment in preventative measures. Emphasis was also placed on understanding relationships across and within the animal and human health systems.

Local researchers conducted interviews in respective national languages. They then transcribed and translated data from audio recordings into English. One author undertook a thematic analysis of the data, identifying themes from the data as to how components of regulatory capacity were reflected.

We refined the preliminary findings of the interviews based on feedback from local researchers and government partners at a September 2019 meeting hosted by the Pasteur Institute in Vietnam.

## 3 Results

### 3.1 Analysis of regulatory capacity

#### 3.1.1 Collaboration across sectors

In all three countries, the Ministry of Health (MOH) and the Ministry of Agriculture (MOA)^1^ are responsible for oversight of the human and animal health sectors, respectively. The JEE [7,24,25] and PVS [[4], [5], [6]] note that collaboration between the animal and human health sectors needs strengthening, across prevention, surveillance, and response activities, and that not even information sharing is systematic across all three countries.

In particular, the JEE and PVS from the three countries suggest that while functional mechanisms for the coordination and integration of sectors into the integration of the IHR have been established, specific mechanisms for responding to zoonotic disease were weaker, with “commitment” differing across sectors in Cambodia (JEE Cambodia), coordination not being operational or information sharing not being timely (JEE Laos), and staff retention (JEE Vietnam) or training (JEE Cambodia) being an issue, in part due to lack of resources for the response. Similarly, the PVS found that coordination at the lower levels between the animal and human health systems in Cambodia was also ineffective (Cambodia PVS).

#### 3.1.2 Relationships within the animal and human health systems

Decentralisation patterns shape relationships within the regulatory structures in the human and animal health sectors in all three countries. For example, the national departments of animal health do not have direct oversight of the plans, budgets, staffing or activities of provincial departments of animal health which report to a separate department in the MOA [[4], [5], [6]]. The PVS from Vietnam notes that with decentralisation, national, provincial, district and commune political levels are heavily involved in decision making, “acting as filters for both policy directives and instructions flowing down the system and information and field technical perspectives flowing upwards” ([6], page 4). Similar dynamics likely impact relationships within the MOH, although they have been slightly more centralised to date. For example, in Cambodia, reporting lines within the health sector were vertical, i.e. through the MOH, until recently when they changed to operating via provincial and district leaders. It is unclear to what extent centralised structures can more easily facilitate regulatory enforcement.

Animal health professionals are less regulated than their human health counterparts. Currently, veterinarians in all three countries are self-regulated through a professional association (although inactive in Cambodia) and are permitted to practice after obtaining the requisite degree [5]. In contrast, practising human health professionals must register with the relevant professional body, and this system is evolving under ASEAN. Village Animal Health Workers (VAHWs) comprise the largest portion of the service delivery workforce in all three countries. In Cambodia and Laos, they are described as volunteers or self-employed agents who derive their income by delivering services on a user fees basis, largely absent of regulatory oversight [4,5].

Relationships between regulators and public providers are shaped by incentives in the human health sector, partially to promote more equitable coverage of services [19,26]. For example, Cambodia is making progress towards universal (human) health coverage using incentive-based policy interventions, such as performance-based financing for subnational authorities, health facilities and/or health workers, combined with more traditional public sector management approaches [19,26]. Additionally, a series of contracting models have been used for the delivery of public primary health care since 1996 [[27], [28], [29]]. Health equity funds, which balance incentives to use services on the part of the population and incentives to provide them on the part of public services, have been implemented since 2000. A midwife incentive scheme has been in place since 2007 to increase facility-based births, and performance-based payment has been used for primary health care since 2008 under the most recent contracting model [30].

In Laos, health equity funds were introduced based on learning from Cambodia, and since 2016 have been merged with three other financing schemes into a single National Health Insurance scheme (first implemented in one Province in 2017 and now covering all 17 provinces excluding the capital, Vientiane). In 2010, Laos adopted the Health Personnel Development Strategy with the central tenet to ensure appropriate incentives for health workers, including providing for rural allowances (introduced in 2015), and allowing public sector doctors to run private clinics in order to help retain them [31,32]. In Vietnam, public hospitals were granted significant autonomy in the 1990s in an effort to increase the incentives to activity, and after this was found to be cost-inflationary, a raft of provider payment reforms, including capitation payments for district hospitals, were introduced to attenuate that [33,34]. Financial incentives have been used to retain staff in rural areas, alongside early promotions to full civil service positions for those who stayed at least three years [35]. In all three countries, these incentive-based policy interventions are combined with more traditional public sector management approaches to achieve public objectives and all three countries have made substantial progress towards Universal Health Coverage. However, understanding human-health-system reforms in all three countries requires caution as they are at different stages and not always working as intended. Also, lessons learned, including difficulties, are not always documented. Similar initiatives are absent for public providers in the animal health sector [4, 5, 6,].

In the veterinary health system, this transition does not seem to have occurred at either the same pace or with the same level of flexibility. Regulations are currently characterised by command and control approaches; public officials are instructed to undertake particular tasks or follow rules, whereas private actors are required to meet standards and conduct business within given parameters, without modifying incentives that may mitigate against the rules set and encourage them to be flouted. To this end, it seems that human and animal health governance currently operates on the basis of conflicting models of how change can be achieved, which could go some way to explaining the current difficulties of achieving collaboration and integrated action under a One Health approach on the ground.

Regulation of private providers in either system is limited. Better regulating and incorporating private providers is considered one of the next steps in health system development, particularly regarding surveillance [[4], [5], [6],^19^,^26^]. The same applies to the animal health sector and is imperative, particularly given that the system predominantly relies upon VAHWs. The numbers of private veterinary practices and VAHWs are shown in Table 2. In Vietnam, some VAHWs receive salaries or fees from the Commune People's Committee (CPC), a local administrative body, donors, or international NGOs, giving them a more mixed public/private character [6].

**Table 2:** Numbers of private animal health workers in Cambodia, Laos and Vietnam.

	Cambodia	Laos	Vietnam
Private veterinary practices	Unknown	6 private but likely increased since 2012 when vets (approx. 26 annually) began graduating from Lao National University	Approximately 1600
Commune or village animal health worker (VAHW) with informal training	12,420 VAHWs working across 14,000 villages • 8% women • 45% active	11,571 VAHWs trained across 11,400 villages. • 12% women • 61% active	30,000 private par- professionals, mostly VAHWs

Note: Values in Laos and Vietnam are from 2010, values for Cambodia are from 2018.

Source: [[4], [5], [6]].

Relationships between regulators and providers are partially characterised by competition in the animal health sector, as some animal health officials play dual roles in regulation and provision. Dual practice is common across both the animal and human health sectors. For example, two-thirds of public employees in the human health sector in Cambodia reportedly work in the private sector [19]. Dual practice persists because there is limited regulation, and raises concerns about performance impacts, and conflicts of interest [5,36]. Concerns over conflicts of interest relating to regulating and participating in medicines sales are particularly pronounced in Laos [4].

Information sharing between the regulatory structure and animal health practitioners is limited. While health information systems and surveillance systems have strengthened within the human health sector in the past decade, animal health information systems are still predominantly paper-based and reliant on information provided by VAHWs. Still, it is unclear how these reports are collated and analysed, with VAHWs reporting a limited understanding of disease outbreaks beyond their respective villages. In Vietnam, the PVS describes VAHWs reporting to the CPC rather than the OAHP, and that CPC officials sign off on their monthly monitoring reports [6].

#### 3.1.3 Relationships with livestock owners

There is little information in the JEE and PVS on the relationships between the regulatory structures, aside from widespread acknowledgement of the limits of their knowledge relating to zoonosis. For example, in Vietnam, where there is a formal communications team within DAH, the JEE found that “farmers, breeders and communities appear to have limited knowledge on risks of zoonotic diseases and measures to reduce inappropriate and at-risk behaviours” ([6], page 14). Similarly, the Cambodian PVS notes that there is minimal interaction and communication between veterinarians and smallholder farmers [5] and the Laos PVS notes that the MOA has ceded control of animal health communication to specific projects [4].

### 3.2 Avian influenza case study

Researchers conducted 44 interviews in Cambodia (*N* = 15), Laos (*N* = 16) and Vietnam (*N* = 13) between June and August 2019. Interviews were conducted with government officials responsible for human and animal health at the national (*N* = 14), provincial (*N* = 5), and district levels (*N* = 12), as well as with poultry owners (N = 13), as shown in Table 3.

**Table 3:** Number of KII by respondent type and country.

	Cambodia	Lao PDR	Vietnam	Total
National	5	2	7	14
Provincial/district	5	9	3	17
Poultry owners	5	5	3	13
Total	15	16	13	44

#### 3.2.1 Relationship between the sectors

Despite the introduction of common guidelines and coordination mechanisms, key informant interviewees described institutional constraints to coordination at the national level, impacting implementation, as shown in Appendix E. The competitive nature of the policymaking and national budgetary allocation processes created tensions between ministries, and respondents suggesting that this impacted collaboration in implementation. All three countries' ministries have different reporting lines, as well as both shared, and sometimes, opposing mandates (e.g., promoting human health and promoting trade). Even with established guidance on joint operations between the health and agricultural sectors (e.g., Circular 16 in Vietnam), the implementation of joint operations has proven difficult. Reflective of these challenges, one national-level official suggested that the interaction between the ministries was limited to the goal of information sharing only, although as discussed below under point 6, this is also difficult in some contexts.

“I have tried to work with X [the other sector]… following the joint response guidelines, but we still cannot work together. So, I decided to push for information sharing only.”

Development partners have supported progress on policy development and coordination at the national level, which interviewees suggest is associated with nascent ownership of the reforms. In Cambodia, several policies (e.g., the Joint SOP and Strategic Plan for Zoonotic Control 2014–2018) were developed with support from donors but not yet officially endorsed. Subnational respondents noted that this had added confusion as to whether the SOP is officially endorsed and should be followed. In Vietnam, the One Health Partnership Committee did not yet have an official function. Its role during a zoonotic disease outbreak remained unclear; respondents also noted that there was limited scope to redress this as there was a ban on new committees chaired by the Prime Minister. As one national-level respondent noted:

“Interdisciplinary coordination is currently primarily supported by donors, international partners under the form of development projects that have not yet been transformed into sustainable activities of each sector. Surveillance to detect early agents of pandemic risk is still a concern of donors and international organisations, not a direct concern of the authorities and national agencies.”

Coordination between national and sub-national levels was largely dependent on non-emergency protocols. For example, in Cambodia, the sub-national level must request support from the national level. At the subnational level, coordination mechanisms between the animal and human health sectors were ad hoc in Cambodia and Laos and dependent on the provincial or district level. Communication took place between human and animal health officials at ad hoc meetings (e.g., those called by sub-national leaders), online messaging apps, or traveling to conduct disease outbreak investigations. In Vietnam, Circular 16 describes establishing an intersectoral committee following the identification of an outbreak, although this circular is not yet institutionalised.

Coordination was also impacted by differences in surveillance systems and financing. Surveillance and laboratory systems were at different stages in the animal and human health systems. Digital information systems in human health are gaining in coverage and functionality while those in animal health were only in the early stages (e.g., the Vietnam Animal Health Information System was currently being piloted) and were primarily activity-based. It is unclear to what degree reporting from VAHWs provides information that may serve as a passive surveillance system and whether anyone collates and analyses these data. There is some active surveillance for AI in humans and animals, but it is ad hoc and largely donor funded. Animal health laboratory capacity is limited; only the national level provides testing for animal health samples in Cambodia and Laos.

In the event of an outbreak notification, policy documents provide for information sharing between the sectors to some degree in all three countries. However, the extent of information sharing between the sectors varies. Respondents provided examples of effective information sharing between human and animal health officials at the sub-national level either directly between officials or via provincial or district leadership at multi-sector meetings or shared social media channels. Conversely, respondents also provided examples of delayed information sharing between human and animal health for notifications of AI. Participants also reported limited information sharing within ministries at the national level. Information was disseminated up but not across sectors or down reporting lines until after an official outbreak declaration. Within the animal health sector, the timing of an official outbreak declaration is sensitive, given that it can result in the movement of animals out of the affected area. For example, one national-level respondent noted:

“X [Ministry] often hides information from us recently. For example, they found Y, but they didn't report the case to us. However, we still got the information from other [donor] partners. In fact, in our SOP [Standard Operating Procedure], X should report/share any information with us immediately after they detect it, but they didn't… X often followed its own bureaucracy. They often wait for official [declaration] from their ministry before sharing the information with us.”

In all three countries, officials reported that public financing for an outbreak response was only available after officially declaring an outbreak. At the sub-national level, officials suggested that this impacted outbreak investigation. They were reliant on donors (including WHO) or personal resources to fund initial investigations (e.g., costs for petrol, per diems, supplies). In one country, this served as a disincentive for sub-national officials to conduct investigations in one sector. Sub-national officials also perceived an imbalance in the resources available to health and animal health, with animal health officials suggesting insufficient resources available for investigations and supporting VAHWs compared to human health volunteers in the community. Officials in two countries suggested that there should be a joint pooled fund to finance outbreak investigations.

#### 3.2.2 Relationship within the systems

Respondents paid specific attention to the capacity of the animal health system and described both supply and demand-side constraints which impact notifications. In one country, health officials described the number of district animal health staff as limited. There was a heavy reliance on VAHWs in each country (see Section 3.1 for a fuller discussion). The most in-depth data on the role of VAHWs from the interviews comes from Cambodia, where poultry owners reported that they were unlikely to seek care for sick birds from VAHWs or make notifications of possible AI to them. Poultry owners preferred to seek care from other providers because VAHWs were inaccessible (phone number not available), unavailable (due to competing demands) or ineffective (without supplies). VAHWs were also negatively associated with the official outbreak response to AI (culling programs). Other poultry owners reported a preference for traditional medicine, self-prescribed medicine or private veterinarians associated with feed companies (as opposed to the VAHWs). Some poultry owners reported knowledge gaps about who to seek care from and how to make a notification. Distance to veterinary services was reported as another factor that may impact notification and was also reported to impact the coverage of preventive services within the animal health sector, e.g., vaccination in Vietnam.

#### 3.2.3 Relationship with livestock owners

The interviews with poultry owners revealed that animal health, food security and livelihoods were ongoing concerns, with officials suggesting that the African Swine Fever outbreak had already led to a change in livestock ownership amongst villagers in one country. However, poultry owners generally saw AI or illness in their poultry flocks as a regular occurrence or “seasonal illness”. As one poultry owner in Cambodia described:

“To be honest, I don't know what H5N1^2^ is, but sometimes I experience almost all of my chickens dying. When it comes to that season of chicken dying, almost every chicken from each household in the village dies. For chicken from family farmers like us, we don't do many things. If they die, we let them die naturally. We never report to the village vet even in the case that many chickens die.”

The exception to this was owners of larger farms in Laos, who have relationships with collectors and vaccinate/have vaccinated birds as part of that relationship.

“When buying poultry, we select from a reliable company such as CP [Charoen Pokphand] where they vaccinate the poultry before sending to farm, and there will be technical staff from the company who train us how to prevent and keep chickens from bird flu. [N]o avian influenza has been found in this area.”

Culling programs operated in all three countries, but Vietnam is the only country to develop a formalised policy compensating owners for mandatory culling. Laos used some contingency funds for HPAI to fund compensation on an ad hoc basis [4].^3^ Cambodia has used emergency funds for control measures (“*movement restriction, isolation, disinfection, treatment, killing, and disposal of animals or animal products”)*, however, farmers do not generally receive compensation because the legislative framework does not provide it [5].

In Cambodia, where there was no compensation for culling, poultry owners (and Village Chiefs) were concerned about the culling of their (and their neighbours') poultry in an outbreak response, which disincentivised reporting. This was exacerbated where poultry owners had taken on debt to finance their poultry farming and were concerned about how they would repay their loans if their poultry were culled. Strategies to avoid culling included not notifying and relocating, hiding, or selling poultry at the market during an outbreak response. Officials and poultry-owner respondents in Cambodia and Vietnam agreed that without cooperation from smallholders and sufficient resourcing, culling programs are challenging for officials to carry out effectively.

### 3.3 Summary of findings

**Fig. 1:**
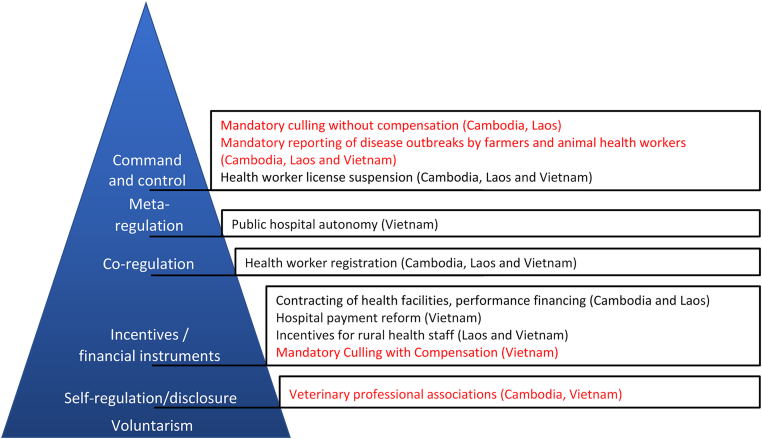
Responsive regulation pyramid with examples of the range of regulatory strategies in the animal (red) and human (black) sector taken concerning notification and response to zoonotic disease outbreaks [37]. (For interpretation of the references to colour in this figure legend, the reader is referred to the web version of this article.)

As summarised in Fig. 1, differing regulatory capacities within the two systems are reflected in the differing range of regulatory approaches adopted across the sectors and countries. All three countries have used incentive-based approaches, including contracting, performance-based financing and other payment reforms, to improve the distribution and quality of human health services amongst public providers. In contrast, the animal health sector has taken very few such approaches [19,26,27,32,33]. Vietnam has incentivised reporting by adopting a mechanism to compensate farmers for the culling of poultry in an outbreak. Cambodia and Laos have not done so.

## 4 Discussion and conclusions

This study revealed continuing challenges to greater collaboration between the animal and human health sectors identified by government officials from both sectors that constrained the implementation of policy responses to outbreaks of AI. Other studies adopting a One Health lens have noted challenges regarding collaboration between the two sectors in the region. For example, Mitchell et al. [8] found that in Vietnam, information was exchanged through informal relationships between officials across human and animal health but there was limited collaboration between the two sectors in conducting investigations about antimicrobial resistance. Similarly, in Sub-Saharan Africa, Okello et al. [38] identified the dominance of larger political considerations in shaping health policy decisions with pandemic threat implications, while official accounts emphasise public good focused and consultative policy development processes.

Findings suggest that both sectors are making progress on establishing the legislative and policy framework for responding to outbreaks of AI. External support partially drives these efforts, while ownership of the reforms is still emerging, which has impacted the extent of collaboration between the sectors in practice. This is consistent with a scoping review of multisectoral collaboration by Bennett et al. [39] who find that “*multisectoral action that has strong external support likely has better access to financial resources, but may suffer from limited local ownership (and hence perhaps low motivation), and conceivably organisational blueprints that do not align with ways of doing business in country*”. Coordination requires a jettisoning of ways of operating that are traditionally wholly vertical and highly hierarchical. To do this, bureaucratic risk-taking that challenges the status quo at lower levels of the hierarchy and using political capital at higher levels would likely be needed. Such risks will not be taken by those without a strong interest in the objectives of the exercise, or ‘ownership’. The situation whereby national level established coordination mechanisms have yet to be integrated into the formal governance arrangements likely illustrates this problem, as does limited emphasis on sub-national coordination, notwithstanding the significant role of provincial and district offices in responding to zoonotic disease outbreaks in the three countries.

The study also found that there is a divergence between the approaches taken concerning *how* to strengthen human and animal health systems. Human health systems now use a set of interventions that combine incentive-based regulation with more traditional command-and-control approaches, attracting increased public investment in all three countries. In contrast, animal health systems have relied almost wholly on command-and-control approaches and have attracted less public investment. There is significant responsibility placed on the animal health sector to contain outbreaks of zoonotic diseases in an environment with a complex and conflicting mix of incentives and limited resources. A better understanding of how veterinary service markets function and organise is thus urgently needed in order to inform the development of context-specific strategies to strengthen these markets' responses towards the achievement of zoonotic disease control.

This study also highlighted the differential level of funding and service delivery organisation and capacities within the two sectors, including both the thin and thick regulatory capacities and core components of the systems (e.g., surveillance and information systems), which also serve as barriers to collaboration and implementation of the existing One Health legislative frameworks. Other studies have also emphasised the imbalance in resources and capacities between the two sectors, suggesting a common dynamic across countries. For example, Machalaba et al. [40], in summarising the discussion at the 2018 Prince Mahidol Award Conference on One Health, reported that “core knowledge and technical skill gaps persist that must be urgently addressed, such as the limited basic veterinary and para-veterinary capacity in many countries…” (page 41).

These observations raise the question, what has caused the regulatory capacity in the human and animal health sector to diverge? One explanation may be that national governments and the international community have given greater priority to the public goods delivered by the human health sector, leading to greater attention to the state's role in governing that sector. This is reflected in the greater emphasis on delivering human-health-related public goods within international cooperation frameworks, such as the Millennium Development Goals (MDGs). Greater donor investment accompanied the MDGs, and has provided an increasing emphasis on aid effectiveness principles, including sustainability. Despite these factors generally supporting an understanding of a divergence between animal and human health regarding the promotion of public interest, privatisation has also played its role in the human health sector in undermining public good production. For example, the International Finance Corporation has consistently promoted private sector led and public-private partnership hospital-based health system models that have restricted resources for primary care and the targeting of resources on the poorest populations [41]. Similarly, hospital autonomy in Vietnam has been accompanied by an increasing stratification of hospital service delivery based on users' ability to pay [42].

In contrast, the role of the animal health sector in delivering public goods remains poorly defined. For example, core services that should be delivered within an animal health system are not defined in the IHR, OIE Animal Codes or elsewhere. In comparison, the MDGs and SDGs define key health services for which governments should, at a minimum, ensure population coverage. Moreover, regional and global trade agreements, which may elicit greater compliance from national governments, are only relevant to export countries (and thus may begin to have greater weight in Vietnam). There is thus a need to better define the animal health sector's public goods or public health functions as a first step towards building the state's regulatory capacity within the animal health system.

The next step may be to develop stronger regulatory approaches in the animal health sector, based on an understanding of incentives within the animal health system and how they can be influenced to improve outcomes. An in-depth analysis of veterinary vaccination services in Cambodia between 1979 and 1996, [18] describes these administrative relationships in the animal health sector through the lens of patrimonial exchange, defined by loyalties to hierarchy and reciprocal obligations, and involving a range of formal incentives such as training opportunities and informal incentives such as gifts. The author describes how frontline veterinary workers shifted their services from prevention to treatment as financing moved from public to private. Payment for services shifted from gifts and training opportunities towards cash, leading to fewer vaccinations. Some competition between VAHWs and the District Office of Animal Health and Production (OAHP) may also have begun to characterise their relationship with the transition to user fees [18].

Similarly, results from Cambodian interview data suggest that there is a need to begin with a greater understanding of the relationship between animal health workers, including VAHWs, and poultry owners. Such an analysis should include both the supply and demand-side factors that impact poultry owners' incentives to seek care for and make notifications of suspected AI and from whom they seek care. The patrimonial relationships described by [18] extend to relationships between villagers and VAHWs. VAHWs are the primary means of communicating with smallholder farmers [5]. While this dynamic often promotes effective engagement where demand for VAHWs' services and incentives align, there are also constraints with reporting disease outbreaks and information systems where incentives are often misaligned. For example, VAHWs' reports may prevent farmers from selling diseased meat [18]. The low market values of poultry suggest limited incentives for VAHWs to apply the training they receive regarding HPAI because of farmers' low willingness to pay for prevention advice and vaccinations [43].

Beyond the analysis within the PVS and the IHR, we are not aware of any specific assessment of the functioning of the veterinary service system encompassing both a supply- and demand-side analysis. This contrasts with the human health sector where there are ongoing qualitative and quantitative assessments, including through the national health accounts, health systems in transition reports and various assessments of constraints concerning the supply and demand for services. This is a gap identified by Coker et al. [44], who notes “there is a need to re-examine how existing systems are structured, resourced, and managed to create synergies between animal and human health and in the process reduce the effect of zoonotic disease burdens”.

This research was a small, exploratory study which makes limited claims to the representation of whole countries or the region. As such, there are limitations of the analysis. For example, there was a small sample of key informant interviews in each country, likely to fall short of comprehensive coverage of all regulatory issues in the three countries. There may be areas in which collaboration and implementation differ from the findings presented here. Additionally, comparisons cannot be made across the three countries on the experience of poultry producers because we interviewed smallholders in Cambodia and Vietnam but commercial farmers in Laos. Our research was conducted before COVID-19, and the situation has likely changed in response to that challenge. Despite these limitations, our study provides novel insights on managing zoonotic disease outbreaks from key stakeholders in the Mekong region.

This study has reinforced the notion that strong responses to zoonotic disease outbreaks are required from both the animal and human health sectors to be contained as early as possible. More optimal responses include the environmental sector, especially for diseases carried by wildlife (e.g., wild birds and AI). In this study, we gained a better understanding of the regulatory capacity of the animal and human health systems, particularly regarding joint responses to AI outbreak. While this study does not find the current level of cross-sectoral collaboration optimal, the ideal level of collaboration to achieve effective early notification and response between the sectors, beyond information sharing, needs further exploration in each context to adapt the current policies and plans in a context-specific manner. One way to overcome these collaborative challenges is through the engagement of stakeholders in a participatory process to ensure the implementation of co-constructed solutions and evaluate their effectiveness and impacts [45]. More broadly, the role of the animal health system relating to public health is also poorly defined within national policy frameworks. By better articulating the potential contribution of the animal health system to public health, there may be increased support for adapting the current coordination mechanisms into the national and sub-national governance structures and strengthening the animal health system to meet these public health objectives through incentive-based regulatory approaches.

## Figures and Tables

**Table:** 

	Cambodia	Laos	Vietnam
Most relevant guiding legislation and policy	Law on Animal Health and Production 2016	Communicable Disease Law	Communicable Disease Control Law 2007
	Joint Standard Operating Procedure (SOP) on AI Outbreak Investigation 2014 (unclear if this has been officially approved)	Law on Livestock Production and Veterinary Matters, 2016 (‘Veterinary Law’)	Animal Health Law 2015
			Circular 16/2013/TTLT-BYT-BNN&PTNT dated 27 May 2013, Circular 7/2016/TT-BNNPTNT dated 31 May 2016
		MOU for sharing information and an SOP/guideline on outbreak investigation and response	
	Strategic Plan for Zoonosis Control in Cambodia 2014–2018 (unclear if this has been officially approved)		
			National Plan for AI Prevention and Control, 2019–2025
	(Note there is no human health related communicable disease law but there are sub decrees and other administrative measures in place.)		
		National Emerging Infectious Diseases – Health Security – Public Health Emergency Action Plan 2016–2020	
National coordination mechanisms	Memorandum of Understanding on Collaboration between the MOH and MAFF on Zoonotic Control and Response (MOU), signed in 2012.	National Coordination for Communicable Disease Control (NCCDC)	National steering committee for epidemic control chaired by MOH (CDC Law).
		One Heath Technical Working Group	National steering committee for prevention and control of animal diseases animal chaired by MARD (Veterinary Law).
	Zoonotic Technical Working Group (ZTWG) between MOH and MAFF and other national and international actors, (TOR annexed to MOU).	Emergency Operations Centre (EOC) co-chaired by animal and human health at the national level.	
			One Heath Partnership, established with donor funding, no official function.
	An Emergency Operation Centre (EOC) has been established.		
			A Public Health Emergencies Operation Centre has been established.
			Focal points within DAH (MARD) and PMD (MOH) responsible for coordinating with each other with respect to prevention and control activities, and to meet every six months to evaluate activities (Circular 16).
National – subnational coordination	Subnational staff must request support from the national level.	N/A or no data available?	Focal points within DAH (MARD) and DOH (MOH) responsible for coordinating with their subnational counterparts.
Subnational coordination mechanisms	No formalised coordination between animal and human health at the subnational level.	Emergency Operations Centre (EOC) co-chaired by animal and human health at the provincial level.	Focal points within subnational DAH and PPMC responsible for coordinating with each other with respect to prevention and control activities, and to meet every six months to evaluate activities (Circular 16).
	The Joint SOP includes a common form that can be used to collect information from villagers.		
		No formal mechanism at the district level.	
			In the event of an outbreak, DAH is to advise the People's Committee to establish an “epidemic investigation and handling team” at the commune level including local representatives from health and veterinary departments (Circular 16)
Financing	Government has an emergency fund, available once an outbreak has been declared (Animal Health and Production Law).	Declaration needed so that financing is available for preventive measures	Emergency financial mechanism for interdisciplinary cooperation (Circular 16)
	No mechanism at subnational level.		
Surveillance and laboratory systems	Two sectors have different information systems. Animal health system largely based on activity reports from VAHW, complied at provincial/district level in electronic or paper-based form.	Two sectors have different information systems.	Two sectors have different information systems. Health Information System (HIS) is established and Vietnam Animal Health Information System (VAHIS) is being piloted (with support from FAO).
		Animal laboratory testing facilities are only available in Vientiane.	
			MARD and MOH have established laboratory networks with protocols for sharing information and specimens within 48 h (Circular 16).
	Donor funded active surveillance for AI in some areas.		
	MAFF and MOH have separate laboratory networks and there is no formal collaboration: MAFF only has laboratory detection capability for AI at the national level; and MOH has four laboratories at the national level.		
Notification systems	Livestock owners must report cases of suspected zoonosis to VAHWs or local authorities in person or via phone. No timeframe is given. Notifications must be passed onto the officer in charge immediately (Animal Health and Production Law).	Livestock owners shall inform the nearest government or village authority immediately upon the detection of any sick or dead animals with unknown causes. No timeframe is given in the law (Veterinary Law)	Households must notify local veterinary agency. Local veterinary agency must report to higher level within 24 h in delta area and 72 h in mountainous areas. (Animal Health Law, Circular 7)
			Health facility must notify health authorities within 24 h (Circular 16).
	MAFF and MOH have separate phone notification mechanisms. MOH has toll free and MAFF use paid phone	Reporting hotline 166 for AI.	
Information sharing	There was some confusion as to the extent to which the MOU provides a basis for information sharing.	Agreement between animal and human health to share information, including diagnostic results within the MOU.	Animal and human health officials, at the national, regional, provincial, regional, district and commune level must share information with each other within 24 h of a suspected case (Circular 16)
	There are no formal arrangements for communication at the sub-national level.		
Declaration of an outbreak	Based on advice from GDAHP, Minister of Agriculture will release a *prakas*, which requires approval of the Prime Minister (Animal Health and Production Law)	Responsibility for declaring an epidemic are decentralised to Chief/Mayor, Provincial Governor or Prime Minister depending on the geographical scale. No timeframe is given in the legislation. (Veterinary Law)	Subnational DAH (MARD) is responsible for communication if animal outbreak; and Provincial Preventive Medical Centre (MOH) if human outbreak. Both must agree on the contents of the communication (Circular 16).
			Level of responsibility depends on number of communes, districts or provinces affected (Animal Health Law)
			Decision must be made within 24 h of receiving request to make a declaration (Animal Health Law)
Response – official	MAFF collects samples and conducts culling within 1-3 km. No compensation.	Veterinary government officials must isolate the animals, conduct laboratory testing and take appropriate control measure (Veterinary Law).	SOP for all activities of the PHEOC have been approved according to ISO.
	MOH establishes temporary health post to conduct health screenings, collect samples, provide information to villagers, working through Village Health Support Group.		When suspected, provincial sub-department of animal health will send notice with district or commune veterinary station to isolate poultry and decontaminate the household.
		Quarantine powers granted by law but specifics governed by regulations. (Veterinary Law).	
			When confirmed, provincial sub-department of animal health will send notice to People's Committee to organise destruction of poultry at household. (Circular 16)
		Culling powers granted by law, but geographical area not defined. If culling takes place, State pledges reasonable compensation (Veterinary Law)	
			Compensation of VND 35,000 (USD 1.50) per poultry destroyed (Decree No.2)
		Health sector responsible for testing and treating patients and disseminating health information.	
			Other activities include (i) human and animal health officials visit households in surrounding area; (ii) monitoring by commune health centre for 14 days; (ii) health promotion (prevention, disinfection of farms) disseminated in local area via loudspeaker; (iv) vaccination (MARD, DAH); and (v) bio-safety measures (MARD, DLP).

**Table:** 

	Cambodia	Laos	Vietnam
National coordination mechanisms	National officials described coordination as challenging in practice due to different mandates, budgets and reporting lines, as well as surveillance and information systems.	Staff rotation can impact coordination and reporting.	Committees are not active enough unless there is an outbreak.
		Mixed views as to how coordination works in practice at all levels with some calling for greater coordination and other greater clarification of roles. Most respondents described animal and human health as working together in a coordinated but separate manner.	Respondents noted that coordination between MARD and MOH is emerging. They noted the need for one coordination mechanism established by decree, but that the government currently does not permit the establishment of new committees chaired by the Prime Minister; one respondent suggested that it could be a Deputy PM.
	These challenges persisted notwithstanding the development of joint SOP.		
	One official noted that after attempted collaboration with the sector had failed, he/she was “pushing for only information sharing” but even that was difficult.		
		Numerous respondents suggested that a continuous standing prevention committee is needed.	
			One official noted that cooperation in the development of policies is not strong, and so it limits coordination in implementation.
National – subnational coordination	Subnational staff were unclear as to the status of SOP and whether staff should be trained on them.	Subnational staff reported national staff participation in response operations as helpful.	No comments made
	Subnational staff reported national staff participation in response operations as helpful and were frustrated when they had little guidance.		
	National staff reported that it was difficult to incentivise subnational staff to launch investigations with limited funds.		
	Perception that MAFF was heavily centralised.		
Subnational coordination mechanisms	District governor may hold regular or ad hoc cross sectoral meetings.	Mixed reports as noted above.	The pilot of Circular 16/2013 found that the diseases prioritised by the two sectors differ, and the resources in the two sectors differ.
	Animal and human health may meet on an ad hoc basis during a response operation, before implementing separate activities.		
			General Department of Preventive Medicine (GDPM) and DAH have been working on guidelines on intersectoral coordination for outbreak management and control for the five priority diseases in Circular 16 with support from WHO, but they not yet finalised.
Financing	Perception that MOH has funds for initial response (from WHO) but MAFF does not.	Perception that MOH has money for per diems but MAFF does not.	The pilot of Circular 16/2013 was found that the resources in the two sectors differ.
	District staff may have to self-fund transport for initial response.	Respondents reported limited funds for petrol, per diem (limited to 35,000 kip/day but insufficient), compensation, staffing, vaccines.	Respondents noted that there is not enough evidence to persuade policy makers of the need to invest in prevention, and that resource allocation only follows an outbreak.
	Reliance on donors for AI program, means that government does not have full ownership.		
Surveillance and laboratory systems	Given limited animal health laboratory capacity, samples are sometimes sent from sub-national to national level via taxi.		There have been some efforts at active surveillance but that the results are often not acted upon. One example of action from the subnational level - e.g.: the health department sent the agriculture department an epidemiological report for Rabies each month so that they could target the vaccination program.
Notification systems	Some poultry owners did not know where to make a notification.	Poultry owners more comfortable reporting to the national level.	One poultry owner reported contacting a private company first, but they could not identify the disease so then he/she contacted the local veterinary station.
	Some owners avoided making a notification as: (i) they were afraid animals will be culled, with no compensation; (ii) loss makes it difficult to repay loans; (iii) it is “cruel” that asymptomatic animals will be culled; and (iv) they feared other villages would “hate us”.	Reporting timeliness is an issue.	
			Three poultry owners reported positive experience of reporting outbreaks to authorities, even though their chickens were culled.
		Perception amongst officials that there is the season of poultry dying.	
		Reporting also problematised because disease is asymptomatic.	
	Some owners also discouraged from notifying VAW based on past lack of action.		
	With respect to broader care seeking behaviours, poultry owners were more likely to seek ongoing care from private vets who were more easily accessible and likely available, visited the owner's home, were less likely to make an official notification of AI.		
	Some owners did not perceive illness as a priority but to be natural (“the season of chicken dying”). Owners with a small number of poultry also didn't think that it was worth reporting.		
Information sharing	At the national level, information sharing about notifications of outbreaks was limited. Trust between MAFF and MOH that they would share information with each other was low. MAFF may wait for official declaration of outbreak (*Prakas*) before communicating with MOH.	No comments made	National respondents reported receiving information from word of mouth, TV or by phone rather than through counterparts within other departments or ministries.
			Even within the same ministry, a respondent reported receiving notification of an outbreak more quickly via from the subnational level, as opposed to ministerial colleagues.
	At the district level, there was evidence of better communication about outbreaks at the between animal and human health officials either directly or via the provincial governor.		
			District health officials reported receiving notification of outbreaks from the animal health department by email and phone.
Risk communication	Some evidence that MAFF does not communicate to villagers until an official *Prakas* is issued. Prior to this, district animal health officials reported conducting spraying and trying to control animal movement.	The district committee will contact the community and those surrounding. The MOH will be part of the team to develop and deliver the key messages to target audience.	No comments made
		District official suggested MAFF should announce outbreak as soon as possible to prevent human mortality and interest of to delay any announcement so that a local response can be initiated before farmers sell, move or hide their poultry, and to avoid impact on economy. Some suggest social media should be used.	
	Tension between MOH's interest to inform as soon as possible to prevent mortality and MAFF's interest to delay so that a local response can be initiated before owner's sell, move or hide their poultry, and to avoid impact on economy.		
Response – official	Poultry owners reported that animal health workers did not give advanced notification of response operation.	If the disease is found, it is reported to the committee for a meeting and to find a solution, working through village authority/chief. Infected chickens were prohibited to move and were seized/buried.	One respondent suggested that the value of compensation was inadequate, and that poultry be classified by those (i) under half a kilogram; (ii) over half a kilogram and (iii) laying eggs.
	Culling programs receive limited support from villagers and officials perceived that it would be easier with a compensation policy.		
			One subnational respondent described how culling remains difficult because of the nature of “backyard” farming, where animals roam free. Requires many officials to carry out the culling programs.
		Compensation for culling is not typically paid.	
		Lack of compensation is a challenge to culling.	
			One respondent noted that health promotion messages are not spread beyond the affected villages “for fear of boycott of poultry meat in surrounding areas.”
	Culling programs were described as thorough in that response teams visit and monitor every household, but respondents perceived that officials were not able to locate poultry that had been hidden. The response teams were large (approx. 30 people) but described as “old” and with only a piece of “wood to chase and hit poultry.” (Interviews)		
	In 2018, MOH and MAFF conducted joint SOP exercises to identify challenges. Decided to separate the task of the two ministries and just report to each other in the end.		
Poultry owner response strategies	If response team comes, then (i) transfer of animals to farms owned by relatives in other areas or (ii) hide in bushes. If “slightly sick” then eat or sell at market. If dead, some sell some to market or some (“when too many die” and “we are lazy”) burn and bury.	Poultry owners hide their chickens.	One sub-national respondent suggested that due to the African Swine Fever outbreak, “a large number” of breeders have shifted from pigs towards poultry. Concern was expressed that they would have less knowledge about caring for poultry/symptoms of AI.
	Owners reported changing business as so many animals died. For example, one farmer changed business from 1000 chickens and 300–400 ducks for sale at market (meat and chicks) to 30–40 chickens for consumption and meat sale in response to previous AI outbreak.		

## Appendix A Key informant interviews with national policy makers: Interview guide

1. What is your position?
2. How long have you been in this position?

There is a different set of questions for interviewees in a policy role (GROUP A) or operational role (GROUP B).

GROUP A: For interviewees primarily in a *policy role*:

I would like to ask you questions about your perspective on the government's current policy relating to infectious disease/avian influenza control

1. Can you describe the onehealth policy?
2. What are the main coordinating mechanisms between the different sectors? Who plays the major role?
3. What is your role in regard to policy relating to infectious disease/avian influenza control?
4. What do you think works well or doesn't work well in relation to policy in relation to avian influenza control (or infectious disease control)?

a. Prompt: extent of cooperation across sectors
b. Prompt: evidence/information available
c. Prompt: information flows
d. Prompt: decision making
e. Prompt: interventions
f. Prompt: communication

5. What do you think could be done to address the policy challenges that you mention above?
6. What are the constraints and opportunities to bringing about these changes?

Now I would like to ask you questions about your perspective on how policy relating to infectious disease/avian influenza control has been developed in the past.

7. What has been your involvement in policy development for infectious disease/avian influenza control?
8. What do you find works well or doesn't work well in policy development for infectious disease/avian influenza control?
9. What do you think could be done to address the challenges in policy development that you mention above?
10. How do you think this project could assist you? What information would you like to receive from the project?
11. Could you refer us to any research or policy documents that you think might be useful for understanding the government's policy relating to AI?

GROUP B: For interviewees primarily in an *operational role*:

3. What has been your involvement in operations for AI control?
4. Can you describe a recent AI control operation that you have been part of (for example, a response to a notification, or monitoring of an outbreak)?
  a. Prompt: What was your role in this situation?
  b. Prompt: How were you informed?
  c. Prompt: What steps did you take?
  d. Prompt: What was the result?
5. What worked well in this AI control operation or other operations that you have been part of/observed?
  a. Prompt: Why?
6. What were the main operational challenges in this AI control operation or other operations that you have been part of/observed?
  a. Prompt: Extent of cooperation across sectors, particularly between health and agriculture
  b. Prompt: Evidence/information available
  c. Prompt: Information flows
  d. Prompt: Decision making
  e. Prompt: interventions
  f. Prompt: Communication
7. What do you think should be done or is being done to address these issues?
8. What are the constraints and opportunities to bringing about these changes you mentioned?
9. How do you think this project could assist you? What information would you like to receive from the project?
10. Could you refer us to any research or policy documents that you think might be useful for understanding the government's policy relating to AI?

## Appendix B Key informant Interviews with local officials: Interview guide

1. What is your position?
2. How long have you been in this position?
3. What is your role in regard to control of infectious disease/avian influenza?
4. Have you ever been involved in actions related to control of a notification or suspected outbreak of AI?
5. If yes, what happened (refer to the most recent notification)?

a. Prompt: what was your role in responding to this notification?
b. Prompt: How were you informed?
c. Prompt: What steps did you take?
d. Prompt: What was the result?

6. Note: If the participant is unable to provide much information regarding this notification, ask about another notification time.
7. How did the local one health committee (or other relevant committee) respond to this notification or suspected outbreak of AI?
8. Prompt: when and how did officials from agriculture and health begin communicating with each other regarding the response to the notification or suspected outbreak?

a. Prompt: How did the committee communicate with poultry farmers/producers?
b. Prompt: How did the committee communicate with the broader community?

9. What went well in responding to this notification or other AI control operations that you have observed?
  a. Prompt: Why?
10. What were the main operational challenges in this AI control operation or other operations that you have been part of/observed?

a. Prompt: Extent of cooperation across sectors, particularly between health and agriculture
b. Prompt: Evidence/information available
c. Prompt: Information flows
d. Prompt: Decision making
e. Prompt: Intervention
f. Prompt: Communication

11. What do you think should / is being done to address these challenges?
12. Can you do anything about these challenges or do you require action to be taken at other levels / by other actors?
  a. Prompt: Which levels?
  b. Prompt: What action? E.g.: Financial, technical or political support
13. How do you think this project could assist you? What would you like to receive information from the project?

## Appendix C KII with farmer representatives – interview guide

1. What is your professional background/experience/education?
2. What is your job/role? How is it related to poultry farming?
3. How long have you been in this position?
4. Describe the activities of farmers/producers in your organisation?
  a. Prompt: what types of poultry?
  b. Prompt: how many?
  c. Prompt: what sort of products?
5. As you know, this research project is about flu-like illnesses in poultry, including avian influenza. Who is responsible for the control of avian influenza in your local area?
  a. Follow up: What contact do you have with this person / agency?
6. Have your farmers/producers received any information/education about avian influenza? Who provided this information? Who would farmers go to for further information?
7. Do your farmers/producers experience challenges in regard to control of avian influenza in their flocks?
  a. Prompt: What are the issues / problems?
8. What are you doing to address these challenges? Can you do anything about them or do you require action to be taken at other levels / by other actors?
9. Have you ever been involved in actions related to control of a notification or suspected outbreak of AI? If yes, what happened?
  a. Prompt: If yes, what happened?
  b. Prompt: How was the AI identified?
  c. Prompt: Were the authorities notified? why? or why not? If yes, how?
  d. Prompt: How did they respond?
  e. Prompt: What other steps did you take?
  f. Prompt: What was the result?

Note: If the participant is unable to provide much information regarding this notification, ask about another notification time.

10. How did the local one health committee (or other relevant committee) respond to this notification?
  a. Prompt: How did the committee communicate with poultry farmers/producers?
11. What went well in responding to this notification?
  a. Prompt: Why?
12. What do you think should be done differently in responding to the next notification?
13. How do you think this project could assist you? What information would you like to receive from the project?

## Footnotes

1 *The Ministry of Agriculture, Forestry and Fisheries (MAFF) in Cambodia, the Ministry of Agriculture and Forestry (MAF) in Laos and Ministry of Agriculture and Rural Development (MARD) in Vietnam.*

2 *H5N1 is a subtype of the HPAI influenza A virus*

3 World Bank support to the government of Laos for the Avian and Human Influenza Control and Preparedness Project from 2006 to 2010 compensated poultry owners for culling, although funding was not sustained. See: http://documents1.worldbank.org/curated/en/734091474501621376/pdf/000020051-20140625234737.pdf
